# First Observation of Wild Double‐Flowered *Rhododendron nivale* With Stamen Petaloidy and Possible Parallel Evolution

**DOI:** 10.1002/ece3.74443

**Published:** 2026-09-29

**Authors:** Xuyan Chen, Shuxing Deng, Zhaoshan Wang

**Affiliations:** ^1^ Research Institute of Forestry Chinese Academy of Forestry Beijing China; ^2^ China Railway Eryuan Engineering Group Co., Ltd Chengdu China

**Keywords:** ABC model, parallel evolution, *Rhododendron*, sky island, stamen petaloidy

## Abstract

Parallel evolution of similar phenotypes under similar selective pressures is often viewed as evidence for natural selection. However, how often such parallel variation occurs in floral traits, and its underlying mechanisms, remain poorly understood—especially in alpine plants. Here we report the first discovery of wild double‐flowered plants in *Rhododendron nivale*, a dwarf shrub of the eastern Qinghai‐Tibet Plateau, and the first record of double flowers in subsection *Lapponica* (small‐leaved, high‐altitude sky‐island shrubs). Naturally occurring stamen petaloidy was found in two geographically isolated populations at high passes in Chagyab County, Tibet (4825 m) and Yajiang County, Sichuan (4650 m), China. Individuals from both populations show nearly identical floral modifications: complete stamen petalody, thickened corolla lobes, abnormal style morphology, and denser capsule scales. The two populations are separated by > 800 km with no possible gene flow, yet they occupy remarkably similar habitats (strong winds, low temperatures, short growing seasons). This parallel variation may suggest independent origins under similar selective pressures. Based on the ABC model of floral development, we hypothesize that the double‐flower phenotype results from altered expression of C‐class genes such as *AGAMOUS*. A plastid (rbcL–matK) phylogeny further shows that the two double‐flowered populations are not each other's closest relatives but instead nest within their respective geographic clades, consistent with independent (parallel) origins. Our findings provide unique material for understanding the molecular mechanisms and environmental drivers of floral homeotic variation. A more systematic investigation of floral development genes across *Rhododendron* is warranted, as such rare mutations may reveal the genetic basis of flower evolution and the role of parallel evolution in generating phenotypic diversity in alpine sky‐island ecosystems.

## Graphical abstract

We report the first discovery of wild double‐flowered populations of the alpine dwarf shrub *Rhododendron nivale* on the eastern Qinghai‐Tibet Plateau. Two geographically isolated populations (> 800 km apart) show nearly identical stamen petaloidy and floral modifications in similar harsh high‐altitude habitats, suggesting possible parallel evolution. This rare natural case provides unique material for understanding floral homeotic variation and possible parallel evolution in woody plants.

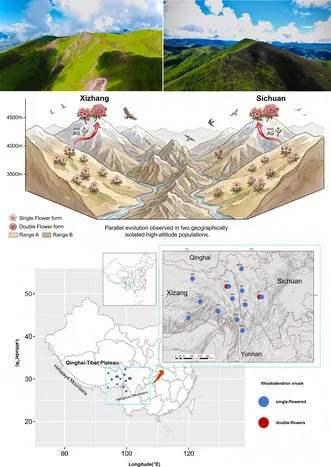

## 1 Introduction

Double flowers resulting from stamen petalody are rare in wild *Rhododendron* populations. To date, only four wild double‐flowered taxa have been documented in this genus of more than 1150 species (Khan et al. 2021; Xia et al. 2021): *R. moupinense*, *R. dauricum* var. *pleni‐florus*, *R. albiflorum* , and *R. ferrugineum* (Rehder 1907; Zhou 2014; Kerner 1865). However, the mechanisms underlying stamen petalody in *Rhododendron* remain poorly understood, and the rarity of such mutations limits comparative studies.

Here we report the discovery of a naturally occurring double‐flowered form of *Rhododendron nivale*, a dwarf shrub widely distributed in alpine scrub of the eastern Qinghai‐Tibet Plateau. During field surveys in August 2023 and August 2025, we encountered two geographically isolated populations of double‐flowered *R. nivale* at high‐altitude passes in Chaya County, Tibet (4825 m) and Yajiang County, Sichuan (4475 m). The two populations are separated by more than 800 km but occur in strikingly similar habitats: windward shaded slopes with strong winds, low temperatures, and a short growing season (Figure 1). Morphological examination revealed nearly identical floral modifications in both populations, including complete stamen petalody, thickened corolla lobes, abnormal style morphology, and denser capsule scales. The later flowering of the double‐flowered morph, compared with the sympatric single‐flowered plants, may reduce temporal overlap and could therefore contribute to reproductive isolation.

**FIGURE 1:**
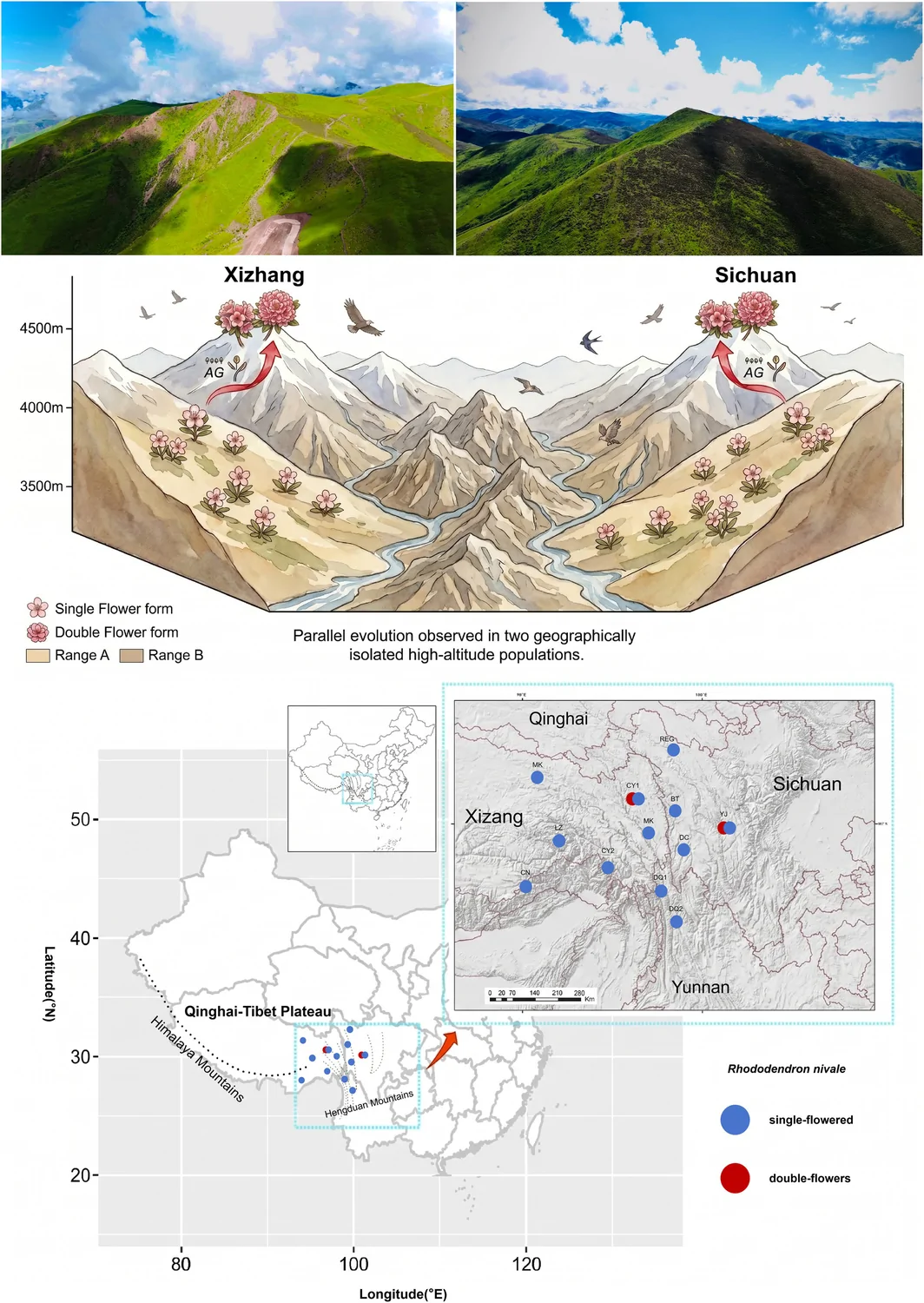
Geographic distribution and schematic illustration of parallel evolution in *Rhododendron nivale* in China. Red dots indicate double‐flowered populations (Chaya, Xizang and Yajiang, Sichuan); blue dots indicate single‐flowered populations. Arrows illustrate the parallel evolution of double‐flowered traits in two isolated populations under similar alpine habitats.

The parallel occurrence of such highly consistent floral anomalies in allopatry suggests independent origins under similar selective pressures—a phenomenon that is consistent with a role for natural selection in driving evolutionary change (Schluter and Nagel 1995; Cai et al. 2019). Based on the ABC model of floral organ development (Kim et al. 2005), we hypothesize that the double‐flowered phenotype in *R. nivale* results from altered expression of C‐class and B‐class MADS‐box genes. Our findings represent the first documentation of double flowers in subsection *Lapponica* and provide unique material for understanding the molecular mechanisms and environmental drivers of floral homeotic variation in alpine plants. Here we report this observation in detail and analyze its adaptation to the alpine environment (parallel evolution) and the mechanism of stamen petalody.

## 2 Materials and Methods

### 2.1 Site Characteristics and Field Works

First discovery and field observation. In August 2023, a biodiversity survey was conducted by the research team in the northern Hengduan Mountains and the upper Lancang River basin, as part of the Second Comprehensive Scientific Expedition to the Qinghai‐Tibet Plateau. At a mountain pass on the border between Kuoda Township, Chaya County, and Gonjo County, Qamdo City, Tibet (30°31′ N, 98°12′ E; 4825 m a.s.l.), we discovered a previously undocumented double‐flowered form of *R. nivale*. The plants grew sympatrically with the normal single‐flowered form on a windward, shaded slope at the edge of *Rhododendron* scrub. Notably, individual branches bore either only double flowers or only normal flowers; no branch was observed to produce both flower types. The population consisted of approximately 30 individuals continuously distributed over the site.

Second discovery and comparative sampling. In August 2025, during a plant ecological resource survey in the Gexigou National Nature Reserve, Yajiang County, Sichuan Province, we again encountered double‐flowered individuals of *R. nivale*. The reserve is located on the right bank of the middle Yalong River, southeastern Qinghai‐Tibet Plateau, and represents a typical forest‐shrub ecosystem of the eastern plateau, with an elevational range from ca. 2800 m (Gexigou valley) to 4702 m (Jianziwan Mountain). The double‐flowered plants were found at a high‐altitude pass at 30°0′25.11″ N, 100°52′36.06″ E, at 4475 m a.s.l., under habitat conditions strikingly similar to those in Tibet: windward shaded slope, edge of *Rhododendron* scrub, mixed with normal individuals. This population comprised approximately 100 double‐flowered individuals, among which a few exhibited transitional floral forms (see Section 3.2).

### 2.2 Morphological Observations and Data Collection

Fresh flowers from double‐flowered and normal plants were examined in the field across multiple populations. Flowers were photographed using Olympus EM‐5 and Canon‐700D cameras. Identification was confirmed by consulting the *Flora of China, Flora of Xizang*, and herbarium specimens (KUN, PE, JSTOR Global Plants). At each site, ten double‐flowered and ten normal individuals were randomly selected. Flowers were fixed in 70% ethanol, and ten flowers per plant were measured under a Leica M205C stereo microscope. Traits recorded included leaf size, pedicel length, calyx and corolla dimensions, stamen number and petalody degree (0–2), style length, and scale density (sparse/medium/dense). Measurements were taken with ImageJ, and means ± SD were calculated per site.

The double‐flowered *R. nivale* was compared with all previously reported wild double‐flowered *Rhododendron* species (*R. moupinense*, *R. dauricum* var. *pleni‐florus*, *R. albiflorum* , and *R. ferrugineum* ; Fang et al. 1999; see also Cai et al. 2022 for R. moupinense), using the same trait set (Table 1). Morphological data were extracted from original publications and JSTOR Global Plants. Statistical analyses were performed in R v4.3.1. Continuous traits were compared between groups using two‐sample *t*‐tests after checking normality (Shapiro–Wilk) and homogeneity of variance (Levene). Categorical traits were analyzed with Fisher's exact test. Significance was set at *α* = 0.05.

**TABLE 1:** Comparison of wild double flowers of five *Rhododendron* species.

Characteristic	*R. nivale* (double‐flowered)	*R. moupinense* (double‐flowered)	*R. dauricum* var. *pleni‐florus*	*R. albiflorum* (double‐flowered)	*R. ferrugineum* (double‐flowered)
First recorded time	July 2023	March 2016	May 2012	July 1904	August 1865
Distribution	Chaya & Gonjo counties, Xizang, China	Wenchuan County, Sichuan, China	Northern Mountain of Moerdaoga, Inner Mongolia, China	Asulkan Valley, Glacier, British Columbia, Canada	Trafoi, South Tyrol, Italy
Altitude	4825 m	1557 m	824 m	1440 m	2212 m
Habitat	Alpine pass	River valley, on rocks	Western hilltop, sunny slope	Mountain forest, near river cliff	Alpine slate scree slope, among shrubs
Flowering period	July–August, later than single‐floPrecocious wered individuals	March, 1–2 weeks later than single‐flowered individuals	May, same as normal individuals	July, later than single‐flowered individuals	August, later than single‐flowered individuals
Calyx	5‐lobed, 2–3 lobes strongly developed, leathery, densely covered with biseriate white scales, margin ciliate	Same as normal form	Same as normal form	Same as normal form	Shorter in double‐flowered form, scaly, margin ciliate
Corolla	Double, 2 whorls, white to pale purple	Double, 3 whorls, white with purplish‐red spots inside	Double, 2–6 layers, pink	Double, 3 whorls, white	Double, 2 whorls, red
Stamens	10, all petaloid, only 2–3 anthers retained at the edge of the outer corolla whorl	Numerous, indefinite number, all petaloid	8, partially or completely petaloid	10 in two whorls, all petaloid	10, all transformed into a funnel‐shaped structure nearly identical to the corolla and fused with it at the base; occasionally, rudimentary anthers in the form of yellowish callosities are visible on this funnel, which corresponds to the stamen whorl
Anther	Partially petaloid, only 2–3 functional anthers remaining, capable of producing normal fruit	Not petaloid	Partially transformed into petals	Completely petaloid; only one case of an incompletely developed anther found	Rudimentary, non‐functional; anthers reduced to yellowish callosities (callus‐like thickenings) on the petaloid funnel structure; no functional anthers present
Filament	Completely petaloid	Lower to middle part transformed into petal‐like structures	Partially transformed into petals	Not petaloid	Completely petaloid
Pistil	Style shortened, not petaloid	Absent (degenerated)	Not petaloid, same as normal form	Carpels transformed into narrow‐elliptic petaloid structures	A cluster of curved, elongated leaflets (petaloid carpels) appearing at the ovary position, green at base, reddish at tip
Other features	Leaves triangular‐cordate, midrib flat; surrounding single‐flowered plants already past bloom	Leaves thickened, margin revolute; does not produce fruit	Fruit subconical, with seeds; trait stable	Surrounding single‐flowered plants already past bloom at the time of discovery	Almost all shrubs at this locality bear double flowers

### 2.3 DNA Phylogenetic Analysis

The plastid barcodes rbcL and matK were sequenced from single‐ and double‐flowered *R. nivale* of Shichuan and Xizang; at least ten flowering and fruiting individuals were sampled per population, using fresh inflorescences and leaves. Sequences of the six reference species were extracted from complete plastid genomes in NCBI GenBank. The rbcL and matK sequences were concatenated into a 1617‐bp matrix of ten taxa, comprising four *R. nivale* accessions and six reference species, the latter including the outgroup *R. anthopogonoides (R. schlippenbachii, R. ambiguum, R. micranthum, R. brachycarpum, R. concinnum)*. Bayesian inference was performed in MrBayes 3.2.7 under the GTR + I + G substitution model, with two independent runs of four chains each, 1,000,000 generations, sampling every 1000 generations and discarding the first 25% as burn‐in. Convergence was assessed by the average standard deviation of split frequencies (ASDSF < 0.01) and potential scale reduction factors (PSRF ≈ 1.0). A 50% majority‐rule consensus tree was summarized, with nodal support reported as posterior probabilities (PP). To complement the Bayesian PP, maximum‐likelihood (K80 + Γ) and maximum‐parsimony bootstrap supports (1000 replicates each) were additionally calculated and are reported alongside PP in Figure 2.

**FIGURE 2:**
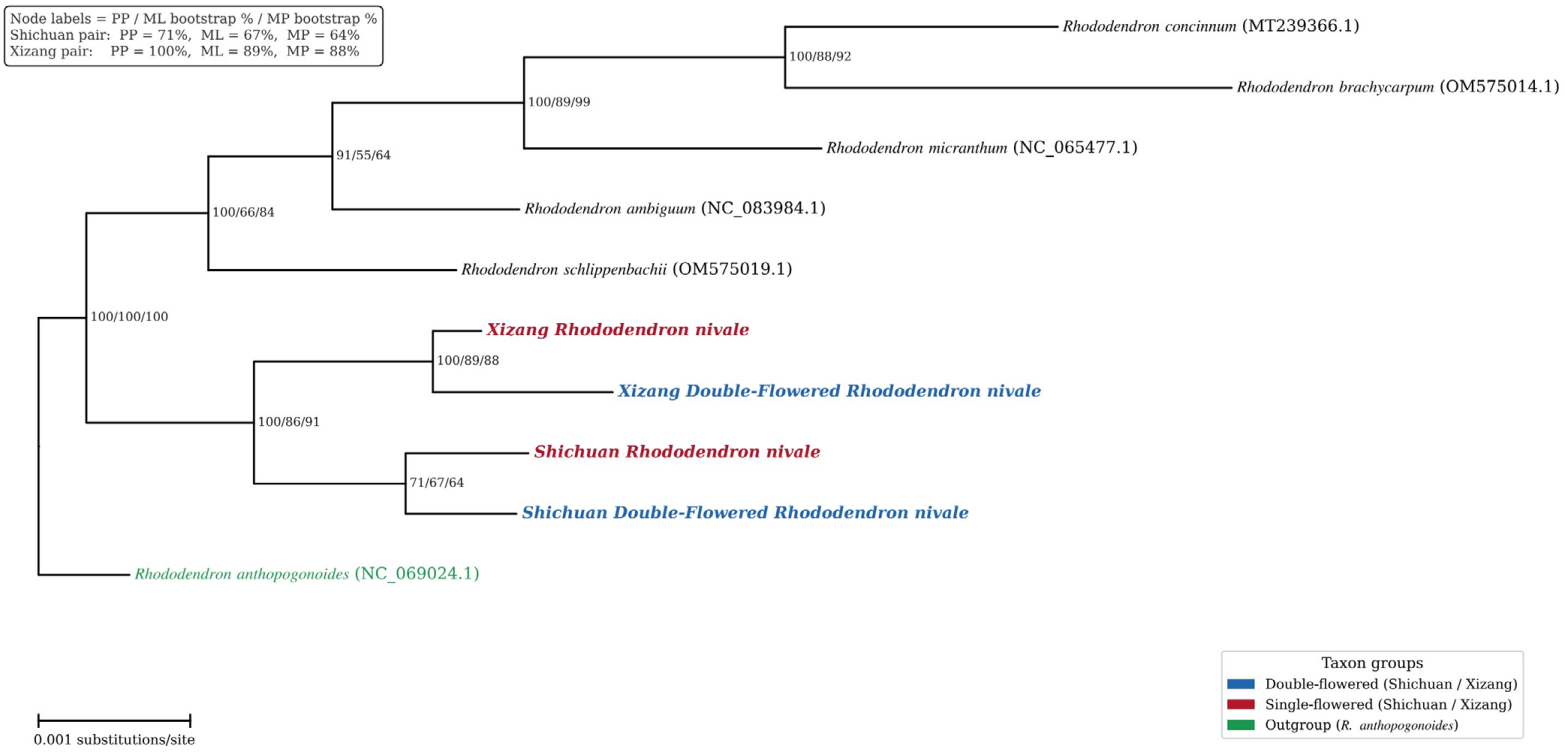
Bayesian majority‐rule consensus tree of Rhododendron based on concatenated rbcL + matK sequences (1617 bp, 10 taxa). The tree was rooted with *R. anthopogonoides* (outgroup). Posterior probabilities (PP), maximum‐likelihood bootstrap (ML, %), and maximum‐parsimony bootstrap (MP, %) are shown at nodes. Double‐flowered accessions are in blue and single‐flowered accessions in red; the outgroup (*R. anthopogonoides*) is in green. The four *R. nivale* accessions form two geographic sister pairs (Shichuan PP = 0.71, ML = 67%, MP = 64%; Xizang PP = 1.00, ML = 89%, MP = 88%). NCBI accession numbers for complete chloroplast genomes are given in parentheses after species names where available.

## 3 Results

### 3.1 Morphological Characteristics of Double‐Flowered *Rhododendron nivale*

The double‐flowered individuals of *R. nivale* from Chaya County, Tibet, and Yajiang County, Sichuan, exhibited highly consistent morphology. Leaves are triangular‐cordate to broadly ovate, 5–6 mm long and 7–8 mm wide, with a flat midrib. The adaxial leaf surface is covered with pale green and whitish scales, while the abaxial surface is densely covered with yellowish‐brown bicolorous scales (Figure 3E–J). Pedicels are approximately 2 mm long. The calyx is 5‐lobed, with 2–3 lobes strongly developed (up to 7 mm long), leathery, and the margin densely covered with two layers of transparent scales. The corolla is pale purple to nearly white; the lobes are 7 mm long, significantly thicker than those of normal plants, and the throat inside is densely pubescent. Stamens are completely petaloid, with only 2–3 residual anthers remaining that produce viable pollen (Figure 4). The style is semi‐globose, 5–7 mm long (16 mm in normal plants), and the base is covered with white sericeous hairs and transparent scales. The capsule is cylindrical, approximately 3 mm long (normal capsule ovoid, 5 mm long), densely covered with two layers of transparent scales (normal capsule with a single layer of white scales) (Figure 5). The flowering period is from August to October, about 1 month later than that of normal plants (May to August) (Table 2). This delay in flowering may lead to possible reproductive isolation between the double‐ and single‐flowered morphs.

**FIGURE 3:**
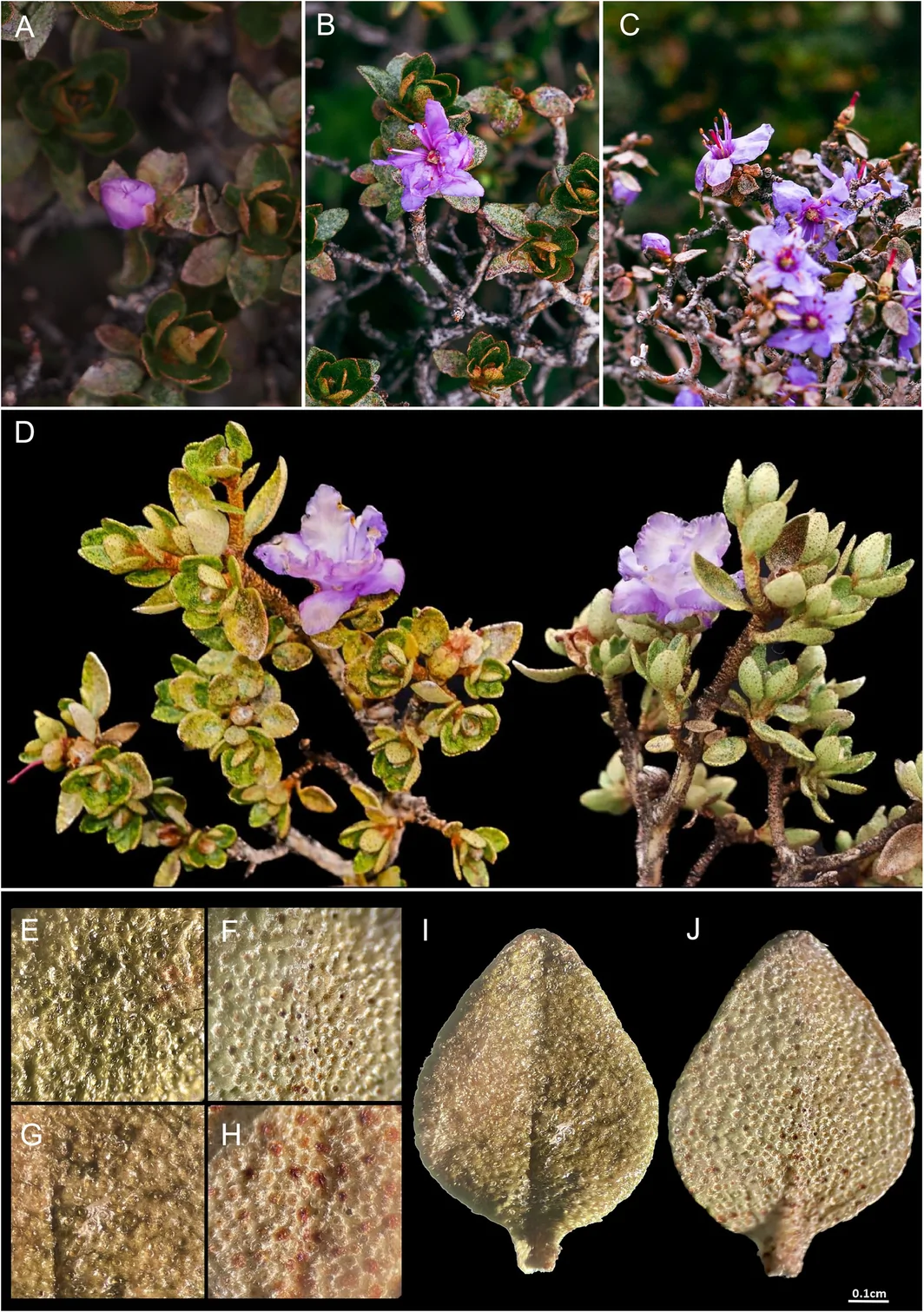
Flower buds, plant morphology, leaves and scale characteristics of *Rhododendron nivale*. (A) Flower bud of a double‐flowered individual; (B) A partially double‐flowered individual from the Sichuan Yajiang population, showing some stamens petaloid while others remain normal (transitional form); (C) A normal single‐flowered plant; (D) A double‐flowered individual from the Tibet Chaya population (fully double flowers); (E) Adaxial leaf scales of a double‐flowered individual (pale green and whitish scales); (F) Abaxial leaf scales of a double‐flowered individual (yellowish‐brown, densely covered with contiguous bicolorous scales, with rufous‐brown scales fewer than adjacent pale yellow scales); (G) Adaxial leaf scales of a normal single‐flowered individual (dark gray‐green, covered with grayish‐white or golden scales); (H) Abaxial leaf scales of a normal single‐flowered individual (yellowish‐green to fawn, with bicolorous pale golden and dark brown scales); (I) Adaxial whole leaf view of a double‐flowered individual; (J) Abaxial whole leaf view of a double‐flowered individual.

**FIGURE 4:**
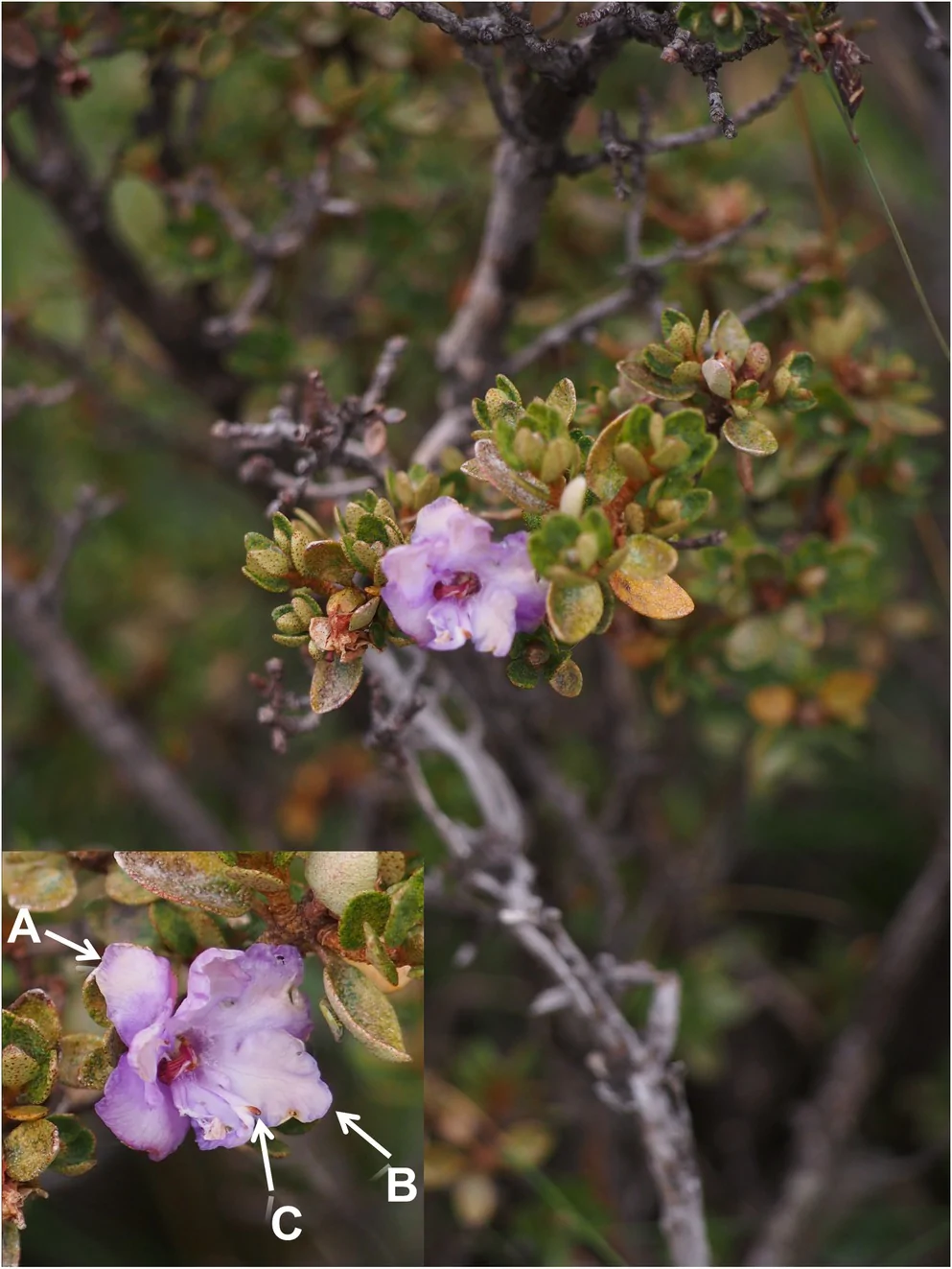
Floral structures of double‐flowered *Rhododendron nivale*. (A) Second whorl petals (outer petals); (B) First whorl petals (inner petals); (C) Remaining anthers (arrowed). (Photos by Xuyan Chen).

**FIGURE 5:**
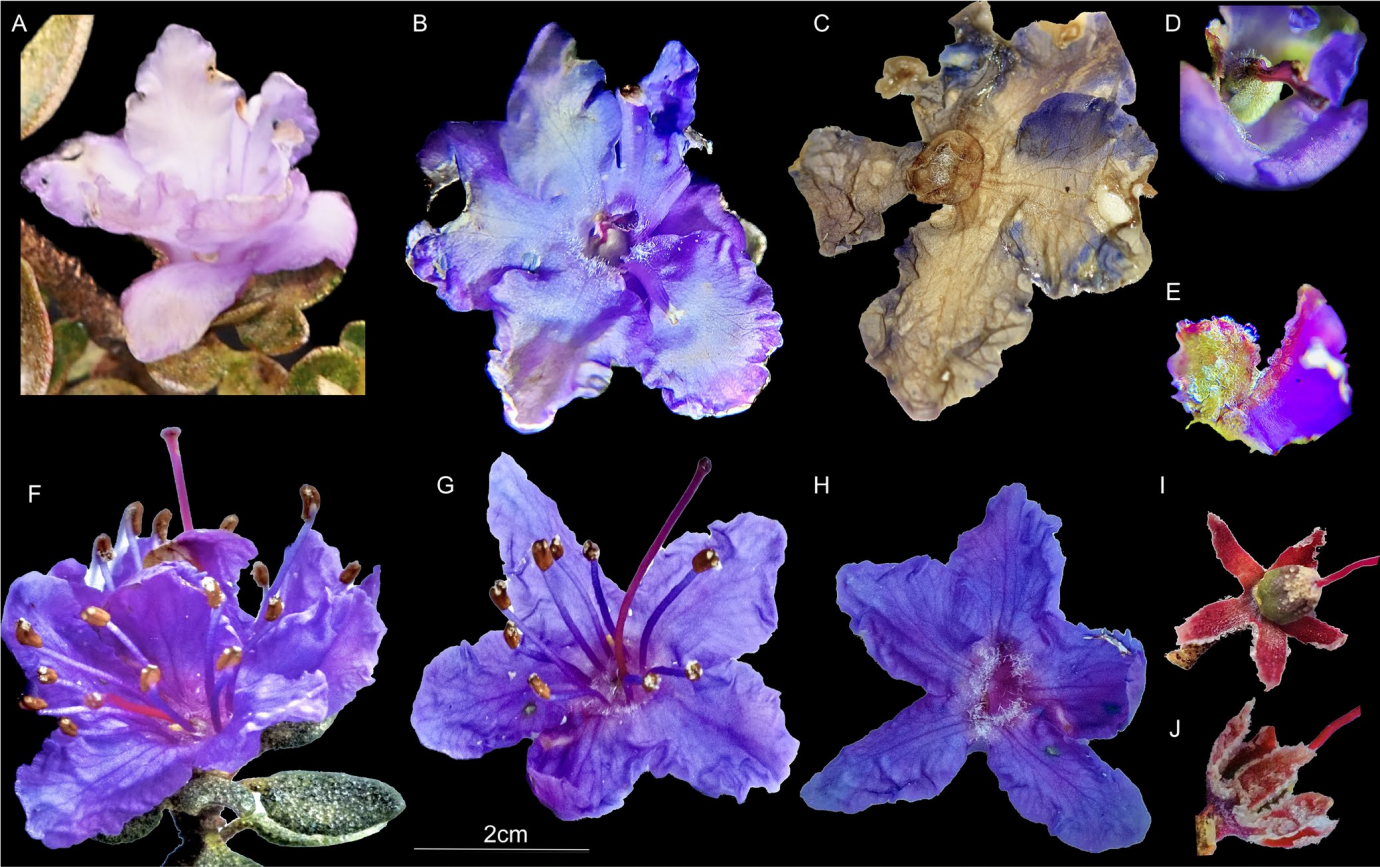
Floral micromorphology of double‐flowered and single‐flowered *Rhododendron nivale*. (A) Whole flower of the double‐flowered form. (B) First‐whorl petals (inner petals) of the double‐flowered form. (C) Second‐whorl petals (outer petals) of the double‐flowered form. (D) Pistil and ovary of the double‐flowered form, showing a shortened style, semi‐globose stigma, and an ovary base covered with white sericeous hairs and transparent scales. (E) Calyx lobes of the double‐flowered form, showing a 5‐lobed calyx with 2–3 lobes strongly developed and leathery, the margins densely covered with two layers of transparent scales. (F) Whole flower of the single‐flowered form. (G) Stamens of the single‐flowered form. (H) Petals of the single‐flowered form. (I) Pistil and ovary of the single‐flowered form, showing a longer style, cylindrical stigma, and a glabrous, smooth ovary with only sparsely scattered scales on the upper portion. (J) Calyx lobes of the single‐flowered form, showing a uniformly 5‐lobed calyx with equal‐sized, papery and elongated lobes bearing a darker central scale band. (Photos by Xuyan Chen).

**TABLE 2:** Morphological comparison between normal single‐flowered and variant double‐flowered individuals of *Rhododendron nivale*.

Characteristics	*Rhododendron nivale* (normal single‐flowered)	*Rhododendron nivale* (double‐flowered variant)
Leaf shape	3.5–9 × 2–5 mm, apex obtuse or rounded, usually without mucro, margin slightly revolute	5 × 7.5 mm, cordate or broadly ovate
Leaf midrib	Conspicuous, slightly impressed adaxially, slightly raised abaxially	Flat, inconspicuous
Leaf scales	Adaxial surface dark gray‐green, with grayish‐white or golden scales; abaxial surface yellowish‐green to fawn, with bicolorous pale golden and dark brown scales	Adaxial surface pale green, with whitish scales; abaxial surface yellowish‐brown, densely covered with contiguous bicolorous scales, rufous‐brown scales fewer than adjacent pale yellow scales
Pedicel	0.5–1.5 mm long, densely covered with dark brown scales	ca. 2 mm long, sparsely covered with reddish‐brown scales
Calyx	All 5 lobes well developed, 4.5 mm long, membranaceous; lobes oblong or strap‐shaped, 2–(−4.5) mm long, outer surface with a central band of scales, occasionally with a few darker scales intermixed among pale scales, margin smooth, eciliate, with a few uniseriate scales only at apex	2–3 lobes strongly developed, remaining 2–3 reduced, up to 7 mm long, leathery; with a narrow band of dark to pale scales from base to apex, margin sparsely ciliate, densely covered with biseriate white scales
Calyx scales	Pale golden, occasionally intermixed with a few dark brown scales; split to near the middle	Scales in two dense layers, transparent to white
Corolla color	Pink to bright purple	White to pale purple
Corolla lobes and tube	Tube 2.5–6 mm long, throat pubescent inside, outside often also pubescent; lobes thin	Tube ca. 7 mm long, throat pubescent inside, margin with dense white pellucid scales, base with imbricate biseriate scales, outside with white pubescence; lobes thicker
Style length	ca. 16 mm, exceeding stamens	5–7 mm
Style apex	Stigma discoid, base glabrous	Stigma semi‐globose, base with white sericeous hairs and white pellucid scales
Stamen number	10	0, all petaloid
Stamen length	ca. 16 mm, filaments villous near base	0
Capsule	Ovoid, ca. 5 mm long	Ovoid to long‐cylindrical, ca. 3 mm long
Capsule scales	Sparsely covered with white scales	Densely covered with biseriate transparent to white scales
Phenology	Fl. May–Aug, fr. Aug–Sep	Fl. Aug–Oct, fr. Oct–Nov
Distribution and habitat	Xizang, Qinghai, Sichuan. Growing in alpine thickets, glacial valleys, meadows, often dominant in *Rhododendron* scrub; 3200–5800 m	Northeastern Xizang. Growing at alpine meadow passes at edge of *Rhododendron* scrub; 4850 m

### 3.2 Transitional Forms

In the Sichuan Yajiang population, we observed clear transitional floral forms: some double‐flowered individuals did not complete full floral homeotic transformation, showing floral morphology intermediate between normal single flowers and fully double flowers; some stamens were petaloid while other stamens remained normal; and pistil morphologies intermediate between the normal and double‐flowered types were also observed (Figure 3A–C). These transitional forms observed in the Sichuan population suggest that the double‐flowered trait is likely controlled by a major genetic factor with incomplete penetrance or variable expressivity. In contrast, no transitional forms were observed in the Tibet Chaya population, where each branch bore either only double flowers or only normal flowers, and no individuals with intermediate floral forms or partially petaloid stamens were found (Figure 3D).

### 3.3 Comparison With Other Wild Double‐Flowered *Rhododendron* Species

Compared with all previously reported wild double‐flowered *Rhododendron* species (*R. moupinense*, *R. dauricum* var. *pleni‐florus*, *R. albiflorum* , and *R. ferrugineum* ), the double‐flowered *R. nivale* is more similar to *R. albiflorum* and *R. ferrugineum* in terms of complete stamen petalody, calyx specialization, and pistil abnormalities, whereas it differs from the partial petalody pattern of *R. moupinense* (Table 1). Notably, the calyx remained unchanged in all cases except in *R. nivale* where 2–3 calyx lobes became enlarged and leathery, representing a novel modification not previously documented in other double‐flowered *Rhododendron*.

### 3.4 Phylogenetic Relationships

Bayesian inference recovered *R. nivale* as a strongly supported monophyletic group (PP = 1.00; ML = 86%; MP = 91%); however, the double‐flowered morph was not monophyletic (Figure 2). The four accessions were resolved into two geographically structured sister pairs: a Shichuan pair (PP = 0.71, ML = 67%, MP = 64%) and a Xizang pair (PP = 1.00, ML = 89%, MP = 88%). Within each pair, the double‐ and single‐flowered morphs were sisters, whereas the two geographic pairs were not sisters to each other. Among the reference species, *R. brachycarpum* and *R. concinnum* formed a well‐supported clade (PP = 1.00; ML = 88%; MP = 92%). These results are consistent with the double‐flowered phenotype having possibly arisen independently in the Shichuan and Xizang lineages of *R. nivale* .

## 4 Discussion

### 4.1 Molecular Mechanism of Stamen Petalody: Insights From the ABC Model

According to the ABCDE model of floral organ development in angiosperms, stamen identity is jointly determined by B‐ and C‐class genes (Kim et al. 2005). Normal expression of C‐class genes (e.g., AGAMOUS) in the third whorl is critical for stamen specification; loss or downregulation of C‐class function can convert stamens into petals (Zhao and Liu 2009). In double‐flowered *R. nivale* , stamens are completely petaloid and the style is abnormal (shortened and pubescent), suggesting that C‐class genes may also affect carpel development. In Arabidopsis, loss of SUPERMAN function leads to extended expression of B‐class genes, resulting in ectopic stamen formation in the fourth whorl (Zhao et al. 2001). However, despite the heuristic value of the ABCDE model, no systematic molecular study has yet been conducted on the genetic basis of double‐flower formation in *R. nivale* . In contrast, recent work in Magnolia polytepala—another basal angiosperm with a multi‐tepal trait—has demonstrated that B‐class MADS‐box genes (e.g., MapoAP3) play a central role in promoting floral organ initiation and identity (Sun et al. 2025). This highlights the urgent need for transcriptomic or whole‐genome resequencing studies comparing double‐flowered and normal Rhododendron plants. By analogy, deficient C‐class expression in the third whorl could redirect stamen fate toward petals. This interpretation is directly supported by molecular evidence double‐flowered Japanese horticultural azalea cultivars (*Rhododendron* spp.), where down‐regulation of the AGAMOUS/PLENA (AG/PLE) homolog together with deletion/frameshift mutations in its coding region caused C‐class function loss and converted stamens and carpels into petaloid organs (Cheon et al. 2017).

### 4.2 Parallel Evolution of the Two Double‐Flowered Populations

The Chaya and Yajiang populations are separated by more than 800 km, yet their double‐flowered individuals show nearly identical floral modifications, and transitional forms occur in both sites. Such striking similarity in allopatry is difficult to explain by gene flow and is consistent with independent origins via parallel evolution. *R. nivale* is widely distributed in the alpine belt of the eastern Qinghai‐Tibet Plateau, and both sites share remarkably similar habitats: strong winds, low temperatures, intense ultraviolet radiation, and a short growing season. Extreme environments may induce stress responses in plant reproductive development, leading to dysregulation of floral development genes. For example, drought or low temperature can affect the expression of flowering time genes (Kooyers 2015) and may also interfere with floral regulatory networks. Moreover, the activity of LTR retrotransposons can be enhanced under environmental stress, causing mutations when inserted near key genes (Galindo‐González et al. 2017). If active transposable elements exist in the genome of *R. nivale* , they might independently generate similar mutations in geographically separated populations. Notably, within each region the double‐flowered morph flowers from August to October, roughly 1 month later than the sympatric single‐flowered plants (May to August; Table 2); this temporal asynchrony may reduce flowering overlap between the two morphs and could constitute a form of prezygotic (temporal) reproductive isolation, which might in turn facilitate the independent establishment and maintenance of the double‐flowered form in the two geographically separated populations.

From an evolutionary perspective, such parallelism, if confirmed, would be consistent with natural selection playing an important role in shaping phenotypes. Wild rice (*Oryza nivara*) originated multiple times from the perennial ancestor *O. rufipogon* across different regions, a classic case of parallel evolution in plants (Cai et al. 2019). Similar parallel evolution has also been documented in sclerophyllous oaks in the East Himalaya‐Hengduan Mountains, where three related species exhibited nearly identical demographic trends under similar selective pressures (Ju et al. 2023). The parallel occurrence of double flowers in *R. nivale* suggests that the harsh alpine environment may exert directional selection on floral architecture. Notably, the stamens in double‐flowered individuals remain fertile, producing viable pollen that can set normal fruit. If double flowers have pleiotropic effects such as attracting specific pollinators or enhancing stress tolerance, they might be maintained in populations. Further studies on pollination ecology and population genetics are required to clarify this.

When similar ecological speciation processes occur simultaneously in different regions, and gene flow between these regions does not significantly affect independent speciation processes, this is termed parallel speciation (Schluter and Nagel 1995; Nosil et al. 2002; Schluter and Conte 2009), a phenomenon that is consistent with a role for natural selection in driving speciation. To date, numerous cases of parallel speciation have been well documented in animals and algae. For instance, threespine stickleback ( *Gasterosteus aculeatus* ) have repeatedly evolved benthic and limnetic ecotypes in isolated postglacial lakes, and the strength of reproductive isolation is closely associated with the degree of parallel morphological and genomic divergence (Roesti et al. 2025; Schluter and Conte 2009). Similarly, the marine snail *Littorina saxatilis* exhibits repeated ecotype differentiation across different geographic regions (Lyu et al. 2015); the land snail *Rhagada* spp. on Rosemary Island, Australia, has independently evolved a keeled‐flat shell morphology on two separate rocky hills, and the ecotypes are maintained by ecologically dependent postzygotic isolation (Stankowski 2013). In algae, parallel adaptive evolution in diatoms has also provided important models for ecological speciation. Furthermore, the measurement of phenotypic parallel evolution has been systematically evaluated, emphasizing that the interaction term in a two‐factor ANOVA or the angle between multivariate trajectories should be used instead of the single‐factor *R* ^2^ metric to accurately quantify parallelism (Arendt et al. 2025). In contrast, parallel speciation in plants remains relatively understudied, and most documented cases are confined to herbaceous species. For example, *Antirrhinum* has undergone multiple independent parallel speciation events between high‐ and low‐altitude ecotypes, involving complex haplotypes and multiple adaptive traits (Barnbrook et al. 2023). Wild rice (*Oryza nivara*) has originated multiple times from the perennial ancestor *O. rufipogon* across different geographic regions, showing parallel adaptation in flowering time and life‐history traits (Cai et al. 2019; MBE.pdf); Qiang et al. (2026) constructed a recombinant inbred line population to dissect the genetic architecture of key adaptive traits, including flowering time and perenniality, between the perennial *O. rufipogon* and the annual *O. nivara*, and demonstrated that loss of photoperiod sensitivity was a major driver of the origin of *O. nivara*, providing genetic evidence for parallel ecological adaptation. In foxtail millet ( *Setaria italica* ), a transposable element insertion in the sh1 gene led to the loss of seed shattering, a change that occurred in parallel across multiple cereal species (Liu et al. 2022). Moreover, independent loss‐of‐function mutations in *GA20ox1* have driven dwarfism and increased silique density in high‐altitude populations of *Arabidopsis thaliana* , representing parallel adaptation to alpine environments (Hou et al. 2025). In fish, the short‐snout icefish ( *Neosalanx brevirostris* ) has independently evolved spring and autumn‐breeding ecotypes in two lakes of the Yangtze River basin, where disruptive selection acting on the magic trait of reproductive season drives parallel sympatric divergence (Yang et al. 2025); the Bahamas mosquitofish (*Gambusia hubbsi*) exhibits parallel behavioral isolation via female resistance under different predation regimes, leading to rapid evolution of reproductive isolation (Pärssinen et al. 2026). Notably, even in woody plants such as Lauraceae, large‐scale diversity analyses have revealed spatial mismatches between functional diversity and phylogenetic diversity, and parallel evolution of morphological characters has been suggested as a possible cause of such decoupling (Zhou et al. 2023). Nevertheless, well‐documented cases of parallel speciation or parallel adaptive evolution in woody plants remain extremely scarce, particularly with respect to complex floral traits. The present study reports the first discovery of natural double‐flower variants in *R. nivale*. This is not only the first documentation of double flowers in subsection *Lapponica*, but more importantly, two geographically isolated populations (over 800 km apart) exhibit nearly identical floral modifications, including complete stamen petaloidy, thickened corolla lobes, and abnormal style morphology. Both populations occur in strikingly similar high‐altitude passes characterized by strong winds, low temperatures, and short growing seasons. Such highly consistent phenotypic divergence in allopatry suggests independent origins via parallel evolution. This discovery provides unique and critical material for understanding the molecular mechanisms and environmental drivers of floral homeotic variation in woody plants.

The Bayesian topology reinforces this inference at the genetic level: double‐flowered *R. nivale* is non‐monophyletic with respect to single‐flowered *R. nivale* , in that within both the Shichuan and Xizang clades the double‐flowered accession is nested as the sister to the sympatric single‐flowered accession, and the two double‐flowered accessions are not each other's closest relatives. This phylogenetic pattern is consistent with the double‐flowered condition having possibly arisen independently, in parallel, in the two regions rather than from a single shared mutation. Consistent with this, the genome of *R. nivale* assembled by Lyu et al. (2024) reveals that this species is genetically specialized for the alpine mountaintop environment with expanded flavonoid and cuticular‐wax biosynthesis gene families, positively selected UV‐repair genes, and significantly upregulated cold and low oxygen responsive transcription factors; autotetraploidy may have further enhanced its high‐altitude survival. These findings suggest that the two allopatric passes may share repeatable, harsh alpine selective pressures capable of repeatedly perturbing the floral‐developmental network, lending plausibility to the parallel origin of the double‐flowered trait; their demonstration that developmental regulatory genes are highly sensitive to environmental signals further supports the notion that environmental cues can modulate floral gene expression. Flowering asynchrony between double‐ and single‐flowered populations reduces overlap, creating temporal isolation that may facilitate independent maintenance of the double‐flowered morph in allopatry. The parallel emergence of the double‐flowered variant in two geographically isolated populations thus suggests that floral homeotic conversion may itself be a product of high‐altitude adaptation—the retained 2–3 residual anthers still yielding viable pollen, with double flowers possibly maintained through altered pollinator attraction or enhanced floral stress tolerance. Collectively, our results offer a new example of repetitive, parallel phenotypic evolution within a widespread species and suggest that double‐flowered morphology across the Hengduan Mountains and Himalaya may be driven by similar selective or developmental forces.

### 4.3 Broader Significance for *Rhododendron* Floral Evolution and Conservation

To date, five wild double‐flowered *Rhododendron* taxa have been reported, belonging to different subgenera or sections, indicating that double flowers may have arisen multiple times independently across the genus. Our discovery of double flowers in subsection *Lapponica* extends the known taxonomic distribution of this rare trait. Most of these double‐flowered occurrences are from high‐altitude or cold regions, suggesting that environmental factors may trigger mutations in floral development genes. Moreover, rapid radiation evolution of *Rhododendron* in the Hengduan Mountains is accompanied by frequent hybridization and gene flow (Ma et al. 2022), which could destabilize gene expression regulatory networks and increase the probability of homeotic mutations.

In addition to genetic mechanisms, environmental signals may also regulate floral development genes. Leaf‐derived signals can affect petal number in Impatiens (Tooke and Battey 2000), and exogenous hormones increase double‐flower frequency (Weijier 1959). Extreme temperatures can cause floral organ increases in *Silene coeli‐rosa* (Lyndon 1979). However, because double‐flowered *R. nivale* , *R. moupinense*, and *R. albiflorum* are found within the distribution ranges of their normal forms and are sympatric or close to them, simple environmental factors are unlikely to be the sole cause; otherwise, double flowers would be more common. Therefore, genetic mutation or abnormal expression is more likely the primary cause, although the involvement of environmental factors cannot be ruled out.

## 5 Conclusion

This study reports the first discovery of natural double‐flowered variants in *R. nivale*, which also represents the first documentation of double flowers in subsection *Lapponica*. The two geographically isolated populations (Chaya and Yajiang) exhibit nearly identical floral modifications, including complete stamen petaloidy, thickened corolla lobes, abnormal style morphology, and denser capsule scales. The presence of transitional forms suggests that the trait is likely controlled by a major genetic factor. Based on the ABC model of floral development, we hypothesize that the double‐flowered phenotype results from altered expression of C‐class and B‐class genes. A plastid rbcL–matK phylogeny suggests that the two double‐flowered populations are not each other's closest relatives but nest within separate geographic clades, consistent with independent (parallel) origins. The striking morphological similarity between the two allopatric populations, combined with their occurrence in similar harsh alpine habitats, suggests independent origins via parallel evolution.

Naturally occurring double‐flowered *Rhododendron* are extremely rare. Due to varying degrees of stamen and pistil petalody and incomplete anther development, sexual reproduction in these plants is inevitably difficult, which also explains their scarcity. Double flowers have high ornamental value; to introduce and domesticate wild double‐flowered *Rhododendron* and preserve this natural variation, further research on its formation mechanism, tissue culture, and artificial propagation is necessary. Future studies should combine genomics, transcriptomics, and functional validation to elucidate the precise molecular basis of stamen petalody and to explore the potential role of environmental factors in triggering such homeotic mutations. The possible parallel evolution suggested here provides a unique system for understanding how floral diversity arises in alpine sky‐island ecosystems.

## Author Contributions

**Xuyan Chen:** conceptualization (equal), data curation (lead), formal analysis (lead), investigation (lead), methodology (equal), writing – original draft (lead), writing – review and editing (equal). **Shuxing Deng:** funding acquisition (lead), supervision (equal). **Zhaoshan Wang:** conceptualization (equal), data curation (equal), funding acquisition (equal), investigation (equal), supervision (equal), writing – original draft (equal), writing – review and editing (equal).

## Funding

This study was supported by the Study on Genetic Diversity of Endemic Poplar Plants in the Himalayas (Grant No. 2019FY100802_04).

## Ethics Statement

The authors have nothing to report.

## Conflicts of Interest

The authors declare no conflicts of interest.

## Data Availability

Data collected for this project is available in Tables 1 and 2 of this manuscript.

## References

[ece374443-bib-0001] Arendt, J. , J. Travis , and D. Reznick . 2025. “On Measurements of Phenotypic Parallel Evolution.” American Naturalist 206, no. 3: 198–205. 10.1086/736845. 40816257

[ece374443-bib-0002] Barnbrook, M. , M. Durán‐Castillo , J. Critchley , Y. Wilson , A. Twyford , and A. Hudson . 2023. “Recent Parallel Speciation in *Antirrhinum* Involved Complex Haplotypes and Multiple Adaptive Characters.” Molecular Ecology 32, no. 19: 5305–5322. 10.1111/mec.17101. PMC10947308 37602497

[ece374443-bib-0003] Cai, S. H. , W. B. Ma , X. Zhou , et al. 2022. “Discovery and Discussion of the Formation Mechanisms of Wild Double Flowers of *Rhododendron moupinense* .” Sichuan Forestry Science and Technology 43: 121–126. 10.12172/202105310001.

[ece374443-bib-0004] Cai, Z. , L. Zhou , N. N. Ren , et al. 2019. “Parallel Speciation of Wild Rice Associated With Habitat Shift.” Molecular Biology and Evolution 36, no. 5: 945–958. 10.1093/molbev/msz021. PMC6501882 30861529

[ece374443-bib-0005] Cheon, K.‐S. , A. Nakatsuka , K. Tasaki , and N. Kobayashi . 2017. “Floral Morphology and MADS Gene Expression in Double‐Flowered Japanese Evergreen Azalea.” Horticulture Journal 86, no. 2: 269–276. 10.2503/hortj.OKD-025.

[ece374443-bib-0006] Fang, R. Z. , H. B. Yang , and C. L. Jin . 1999. Flora of China. Vol. 57, Part 1, 52. Science Press.

[ece374443-bib-0007] Galindo‐González, L. , C. Mhiri , M. A. Grandbastien , and F. D. Deyholos . 2017. “LTR‐Retrotransposons in Plants: Engines of Evolution.” Gene 626: 14–25. 10.1016/j.gene.2017.04.051. 28476688

[ece374443-bib-0008] Hou, X. H. , Y. C. Xu , T. Sun , et al. 2025. “Green Revolution Gene Drives Adaptation of *Arabidopsis* to the Extremely High Altitude.” Science China Life Sciences 68, no. 3: 859–870. 10.1007/s11427-024-2678-9. 39856442

[ece374443-bib-0009] Ju, M. , J. Yang , M. Yue , and G. Zhao . 2023. “Speciation Patterns of Related Species Under the Hybrid Zone: A Case Study of Three Sclerophyllous Oaks in the East Himalaya‐Hengduan Mountains.” Molecular Ecology 32, no. 16: 4610–4626. 10.1111/mec.17060. 37395683

[ece374443-bib-0010] Kerner, A. 1865. “Gefüllte Alpenrosen und Gefülltes Edelweiss.” Oesterreichische Botanische Zeitschrift 15: 285–286.

[ece374443-bib-0011] Khan, G. , J. Nolzen , H. Schepker , and D. C. Albach . 2021. “Incongruent Phylogenies and Their Implications for the Study of Diversification, Taxonomy, and Genome Size Evolution of *Rhododendron* .” American Journal of Botany 108: 1957–1981. 10.1002/ajb2.1747. 34668570

[ece374443-bib-0012] Kim, S. , J. Koh , M. J. Yoo , H. Kong , and H. Ma . 2005. “Expression of Floral MADS‐Box Genes in Basal Angiosperms: Implications for the Evolution of Floral Regulators.” Plant Journal 43: 724–744. 10.1111/j.1365-313X.2005.02487.x. 16115069

[ece374443-bib-0013] Kooyers, N. J. 2015. “The Evolution of Drought Escape and Avoidance in Natural Herbaceous Populations.” Plant Science 234: 155–162. 10.1016/j.plantsci.2015.02.012. 25804818

[ece374443-bib-0014] Liu, H. , X. Fang , L. Zhou , et al. 2022. “Transposon Insertion Drove the Loss of Natural Seed Shattering During Foxtail Millet Domestication.” Molecular Biology and Evolution 39, no. 6: msac078. 10.1093/molbev/msac078. PMC9167939 35388422

[ece374443-bib-0015] Lyndon, R. F. 1979. “Aberrations in Flower Development in Silene.” Canadian Journal of Botany 57, no. 3: 233–235. 10.1139/b79-031.

[ece374443-bib-0016] Lyu, H. M. , R. C. Zhou , and S. H. Shi . 2015. “Recent Advances in the Study of Ecological Speciation.” Biodiversity Science 23, no. 3: 398–407. 10.17520/biods.2015068.

[ece374443-bib-0017] Lyu, Z. Y. , X. L. Zhou , S. Q. Wang , et al. 2024. “The First High‐Altitude Autotetraploid Haplotype‐Resolved Genome Assembled (*Rhododendron nivale* subsp. *boreale*) Provides New Insights Into Mountaintop Adaptation.” GigaScience 13: giae052. 10.1093/gigascience/giae052. PMC11304948 39110622

[ece374443-bib-0018] Ma, Y. , J. Liu , C. Xie , and Y. Li . 2022. “Pervasive Hybridization During Evolutionary Radiation of *Rhododendron* Subgenus Hymenanthes in Mountains of Southwest China.” National Science Review 9, no. 12: 50–60. 10.1093/nsr/nwac076. PMC9844246 36687562

[ece374443-bib-0019] Nosil, P. , B. J. Crespi , and C. P. Sandoval . 2002. “Host‐Plant Adaptation Drives the Parallel Evolution of Reproductive Isolation.” Nature 417, no. 6887: 440–443. 10.1038/417440a. 12024213

[ece374443-bib-0020] Pärssinen, V. , K. Hulthén , R. B. Langerhans , and P. A. Nilsson . 2026. “Divergent Ecological Adaptation in Allopatry Leads to Behavioral Isolation Through Female Resistance.” BMC Ecology and Evolution 26: 23. 10.1186/s12862-026-02345-1. PMC12973862 41772428

[ece374443-bib-0021] Qiang, C.‐G. , H.‐X. Zhang , Q.‐L. Meng , et al. 2026. “Genetic Architecture of Flowering Time and Perenniality: Implications for the Origin of Wild Rice *Oryza nivara* .” New Phytologist 249: 3120–3136. 10.1111/nph.20345. 41457367

[ece374443-bib-0022] Rehder, A. 1907. “ *Rhododendron albiflorum* With Double Flowers.” Botanical Gazette 43, no. 4: 281–282.

[ece374443-bib-0023] Roesti, M. , J. S. Groh , F. C. Jones , C. L. Peichel , and D. Schluter . 2025. “A Species Interaction Kick‐Starts Ecological Speciation in Allopatry.” Proceedings of the National Academy of Sciences of the United States of America 122, no. 42: e2506625122. 10.1073/pnas.2506625122. PMC12557528 41082661

[ece374443-bib-0024] Schluter, D. , and G. L. Conte . 2009. “Genetics and Ecological Speciation.” Proceedings of the National Academy of Sciences of the United States of America 106, no. Supplement 1: 9955–9962. 10.1073/pnas.0901264106. PMC2702799 19528639

[ece374443-bib-0025] Schluter, D. , and L. M. Nagel . 1995. “Parallel Speciation by Natural Selection.” American Naturalist 146, no. 2: 292–301. 10.1086/285799.

[ece374443-bib-0026] Stankowski, S. 2013. “Ecological Speciation in an Island Snail: Evidence for the Parallel Evolution of a Novel Ecotype and Maintenance by Ecologically Dependent Postzygotic Isolation.” Molecular Ecology 22: 2726–2741. 10.1111/mec.12289. 23506623

[ece374443-bib-0027] Sun, L. , Y. Chen , T. Nie , et al. 2025. “B‐Class Floral Homeotic Gene MapoAPETALA3 May Play an Important Role in the Origin and Formation of Multi‐Tepals in Magnolia Polytepala.” Horticultural Plant Journal 11, no. 2: 891–905. 10.1016/j.hpj.2023.08.004.

[ece374443-bib-0028] Tooke, F. , and N. H. Battey . 2000. “A Leaf‐Derived Signal Is a Quantitative Determinant of Floral in Impatiens.” Plant Cell 12, no. 10: 1837–1847. 10.1105/tpc.12.10.1837. PMC149123 11041880

[ece374443-bib-0029] Weijier, J. 1959. “The Interaction of Gibberellic Acid and Indoleacetic Acid in Impatiens.” Science 129: 896–897. 10.1126/science.129.3353.896. 13635030

[ece374443-bib-0030] Xia, X. M. , M. Q. Yang , C. L. Li , et al. 2021. “Spatiotemporal Evolution of the Global Species Diversity of *Rhododendron* .” Molecular Biology and Evolution 39, no. 1: msab314. 10.1093/molbev/msab314. PMC8760938 34718707

[ece374443-bib-0031] Yang, H. , Y. L. Li , T. F. Xing , and J. X. Liu . 2025. “Parallel Sympatric Divergence Driven by Disruptive Selection Acting on a Magic Temporal Isolation Trait in a Wild Annual Fish.” Molecular Ecology 35, no. 1: e70224. 10.1111/mec.70224. 41451577

[ece374443-bib-0032] Zhao, D. , Q. Yu , and M. Chen . 2001. “The ASK1 Gene Regulates B Function Gene Expression in Cooperation With UFO and LEAFY in *Arabidopsis* .” Development 128, no. 14: 2735–2746. 10.1242/dev.128.14.2735. 11526079

[ece374443-bib-0033] Zhao, Y. Q. , and Q. L. Liu . 2009. “Research Advances in the Formation Mechanism and Genetic Characters of Double Flowers.” Acta Botanica Boreali‐Occidentalia Sinica 29, no. 4: 832–841.

[ece374443-bib-0034] Zhou, M. 2014. “Rare Dauricum a New Variant—Plena Dauricum.” Territory & Natural Resources Study 1: 84–85.

[ece374443-bib-0035] Zhou, R. , X. Ci , J. Hu , et al. 2023. “Transitional Areas of Vegetation as Biodiversity Hotspots Evidenced by Multifaceted Biodiversity Analysis of a Dominant Group in Chinese Evergreen Broad‐Leaved Forests.” Ecological Indicators 147: 110001. 10.1016/j.ecolind.2023.110001.

